# Bi-parametric MRI of the Diaphragm Using Dynamic and Static Images: The Initial Experience

**DOI:** 10.7759/cureus.61446

**Published:** 2024-05-31

**Authors:** Murat Tepe, Ibrahim Inan, Safiye Kafadar

**Affiliations:** 1 Radiology, Mediclinic City Hospital, Dubai, ARE; 2 Radiology, King's College Hospital, Dubai, ARE; 3 Radiology, Harran University, Şanlıurfa, TUR

**Keywords:** diaphragm dysfunction, eventration of diaphragm, conventional fluoroscopy, magnetic resonans imaging, idiopathic diaphragm paralysis

## Abstract

Background: With recent technological advances, magnetic resonance imaging (MRI) has offered new sequences that can evaluate the real-time motion of anatomic structures. This study aims to evaluate the interobserver agreement in the diagnosis of diaphragmatic dysfunctions using bi-parametric MRI, in which dynamic sequences for diaphragm movement and static sequences for soft tissue resolution are used together to provide a visualization of the diaphragm.

Methodology: Twenty-nine cases that underwent a bi-parametric magnetic resonance examination which includes coronal T2 single-shot turbo spin echo and the coronal SENSE single-shot balanced turbo field echo real-time sequences were retrospectively evaluated. The images of the patients were assessed by two independent observers. Cohen’s kappa coefficient was calculated to evaluate the interobserver agreement.

Results: The mean age of the patients was 44.86 ± 17.57, ranging from 18 to 80 years. The kappa value was calculated as 0.889, indicating a strong agreement between the interobservers.

Conclusions: Our experience suggests that bi-parametric MRI is a promising tool in the evaluation of diaphragmatic abnormalities.

## Introduction

The diaphragm is a crucial respiratory muscle that separates the thoracic and abdominal cavities, playing a vital role in ventilation. Comprised primarily of striated muscle fibers, it contracts and relaxes to facilitate breathing. Dysfunction of the diaphragm can manifest as dyspnea and respiratory distress, significantly impacting patients' quality of life. Various etiologies, including trauma, tumoral invasion, surgery, and infection, can lead to diaphragmatic dysfunction [1].

Traditionally, the fluoroscopic sniff test has been the gold standard for evaluating diaphragmatic dysfunction due to its high temporal resolution, allowing real-time monitoring of diaphragm movement during spontaneous and deep breathing. This test is particularly useful in distinguishing diaphragm dysfunction, such as diaphragmatic paralysis and weakness, from conditions like pulmonary atelectasis and eventration of the diaphragm. The presence of paradoxical motion in the elevated diaphragm during the sniff test is diagnostic for diaphragmatic paralysis [1]. However, fluoroscopy has significant drawbacks, including exposure to ionizing radiation and limited soft tissue resolution, which hinders the identification of underlying etiologic factors even when diaphragmatic paralysis is diagnosed.

Recent advancements in magnetic resonance imaging (MRI) technology have introduced new sequences that offer high soft tissue resolution and the ability to evaluate real-time motion. Cardiac MRI, for example, utilizes high-temporal resolution sequences to effectively assess cardiac movements and myocardium [2]. Similar sequences can be adapted for real-time evaluation of diaphragm movements [3,4]. Moreover, MRI sequences with high temporal resolution can directly observe diaphragm movement during breathing, overcoming the challenge of artifacts in abdominal imaging. The combined use of conventional MRI sequences with high soft tissue contrast is particularly advantageous in elucidating the etiology of diaphragm pathology. The MRI protocol including the combination of CINE MRI and conventional T2-weighted sequences is referred to as "bi-parametric MRI" in this study.

In the context of respiratory disorders, diaphragmatic dysfunction is not limited to paralysis. Conditions like chronic obstructive pulmonary disease (COPD) and idiopathic pulmonary fibrosis (IPF) also involve significant diaphragmatic involvement [5]. Additionally, ultrasound evaluations in IPF patients have revealed significant differences in diaphragmatic function compared to healthy controls, highlighting the importance of advanced imaging techniques in managing various pulmonary diseases [6].

Furthermore, the incidence of diaphragmatic dysfunction in acute stroke patients is notably high, particularly on the side contralateral to the brain lesion. This dysfunction can be effectively monitored using imaging techniques, which are crucial for early intervention and management [7]. These findings underscore the potential of bi-parametric MRI to provide a comprehensive evaluation of diaphragmatic dysfunction by combining dynamic and static sequences.

This study aims to evaluate the interobserver agreement in diagnosing diaphragmatic dysfunctions using bi-parametric MRI. This approach integrates dynamic sequences for diaphragm movement with static sequences for soft tissue resolution, offering a comprehensive visualization of the diaphragm. By providing a high-resolution and dynamic assessment, bi-parametric MRI has the potential to enhance diagnostic accuracy and offer valuable insights into the underlying etiologies of diaphragmatic dysfunctions.

## Materials and methods

Twenty-nine cases that underwent a bi-parametric MRI examination between January 2017 and May 2018 to evaluate diaphragm dysfunction were included in this study. At the beginning of the study, the ethics committee approval was obtained. Patients’ informed consent was obtained before all the image acquisitions.

Philips Achieva 1.5 Tesla MRI scanner with a 16-channel torso coil (Philips Healthcare, Best, The Netherlands) was used. The MRI protocol consisted of two main sequences; the first was a static sequence consisting of coronal T2 TSE (Single-Shot Turbo Spin Echo) due to its potential diagnostic value on the illumination of the underlying cause of the diaphragm pathology if exists, and the second was the coronal oblique sBTFE-RLT (Sense Balanced Turbo Field Echo Real-Time) dynamic sequences in which the diaphragm movement can be evaluated. The acquisition parameters are detailed in Table 1.

**Table 1:** Image acquisition parameters.

	T2 TSE (Single-Shot Turbo Spin Echo)	sBTFE-RLT (Sense Single-Shot Balanced Turbo Field Echo Real-Time)
TE (msec)	80	1
TR (msec)	750	2
Slice thickness (mm)	7	30
Slice gap (mm)	0.5	-
Flip angle	90	50
Slice orientation	coronal	coronal oblique
FOV (mm x mm)	400 x 400	320 x 302
Matrix	224 x 195	88 x 83
Dynamic scan time (msec)	-	0.115
Total scan duration (sec)	12.7	23.30
Reconstruction matrix	352	144

**Figure 1:**
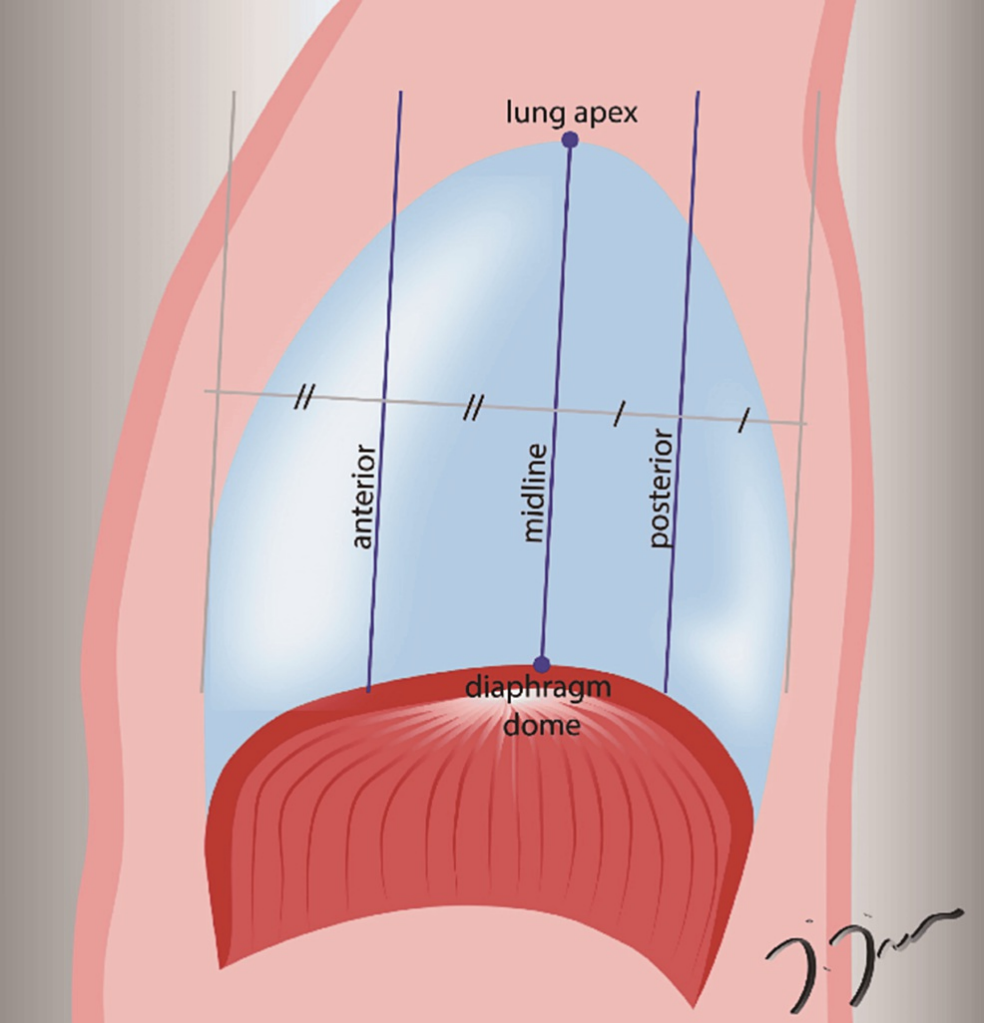
An illustration demonstrating the three coronal oblique planes (anterior, midline, and posterior) used in the dynamic sBTFE-RLT (Sense Balanced Turbo Field Echo Real-Time) sequence. The drawing was created by one of the authors of this article, İbrahim İnan.

Dynamic imaging of the sBTFE-RLT sequences was obtained on three coronal oblique planes dividing the diaphragm into four parts on the sagittal plane (anterior, middle, and posterior lines). The middle line was positioned to pass through the apex of the lung and the highest point of the diaphragm, and the anterior and posterior lines were placed parallel to the middle line. These planes are illustrated in Figure 1.

In addition, dynamic images were obtained in three stages. The first stage was spontaneous breathing in which images are obtained during normal breathing without giving any verbal commands to patients before image acquisition. In the second stage, image acquisition was obtained during deep breathing through the mouth, and the patients were commanded verbally “Take a deep breath through your mouth and release it as deep as possible, and repeat it till the stage was over with another command.” In the third and the last step, the images were taken during a sniff maneuver. A verbal command was given to the patients as “take a very quick and deep breath through your nose with a closed mouth,” and it was repeated a few times for each patient. Before the study, all patients were informed about these stages, and breath maneuvers were practiced a few times. The total acquisition time for the two MRI sequences, which include both static and dynamic scans, was approximately five minutes.

The bi-parametric MRI images obtained from the cases were retrospectively and independently evaluated by two radiologists with 6 and 10 years of experience in diagnostic radiology and MRI. They assessed the diaphragm motions visually at the PACS station and the diagnosis of the observers was noted for each patient. The following criteria were used for the diagnosis of diaphragmatic dysfunction. In cases of diaphragmatic elevation, paradoxical movement of the diaphragm during inspiration on the affected side suggests paralysis; reduced movement in the normal direction compared to the opposite side indicates weakness; normal diaphragmatic movement accompanied by partial or complete lung collapse points to elevation secondary to atelectasis; and focal bulging of the diaphragm contour indicates eventration [1].

Descriptive statistics were expressed as numbers and percentages, and parametric numerical data were presented as mean and standard deviation values. IBM SPSS Statistics for Windows, Version 21 (Released 2012; IBM Corp., Armonk, New York, United States) software was used for statistical analysis. Cohen’s kappa coefficient was calculated to evaluate the interobserver agreement.

## Results

**Figure 2:**
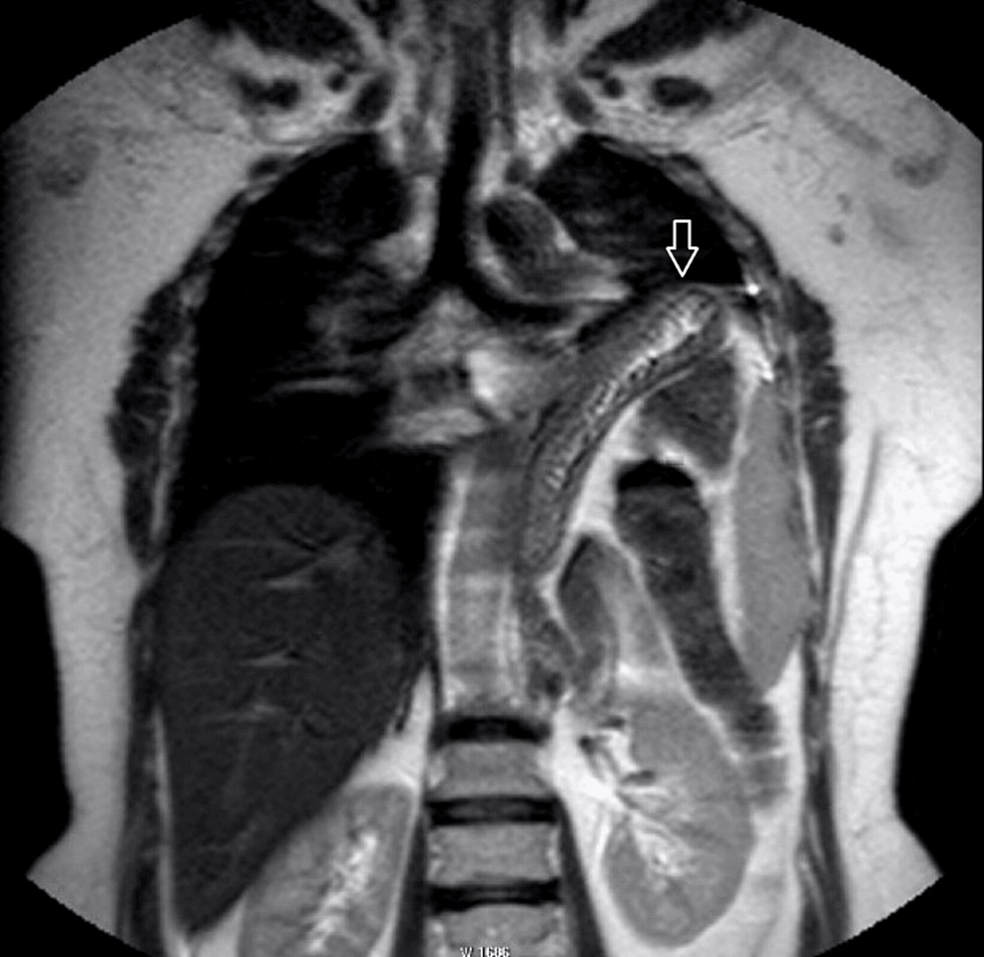
A 54-year-old woman with left diaphragm paralysis. The coronal T2 TSE image shows an elevated left diaphragm (arrow). TSE: Turbo Spin Echo

**Figure 3:**
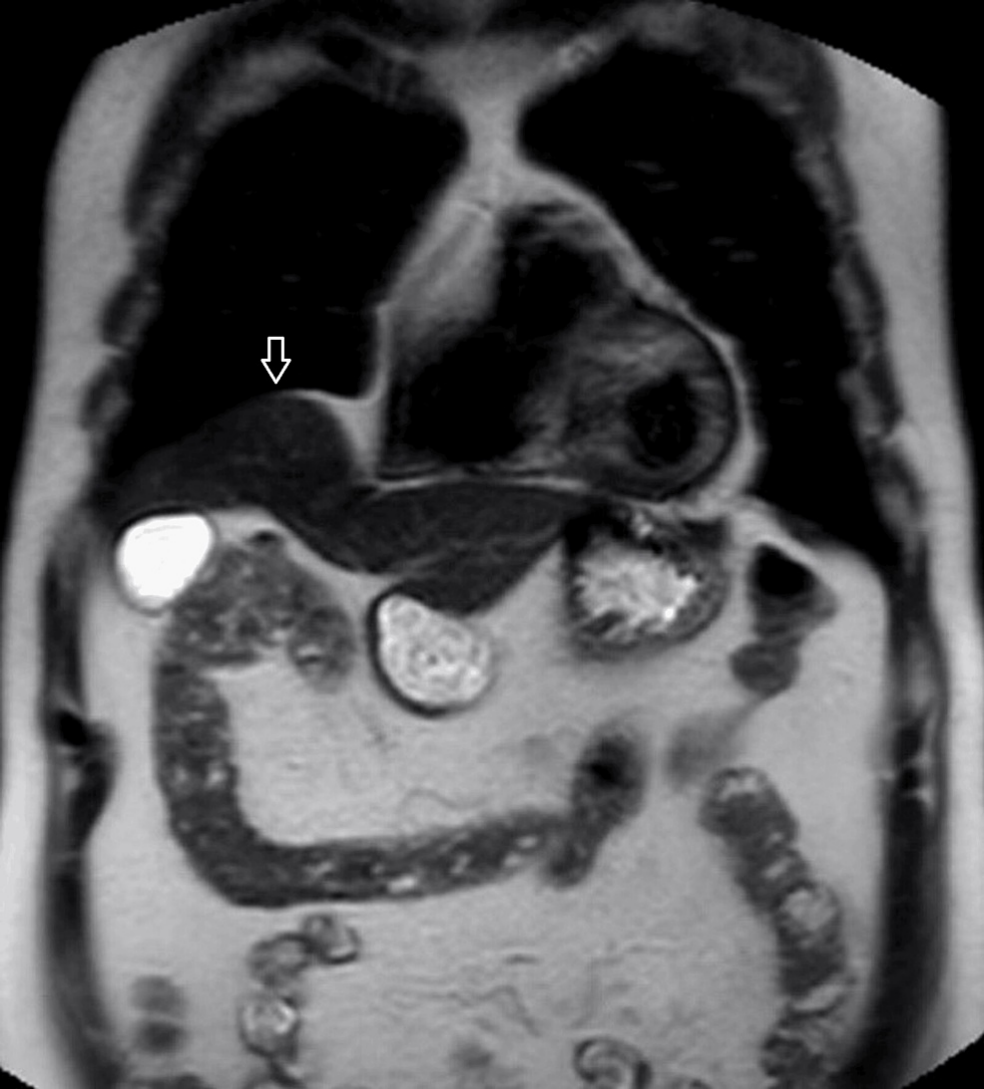
A 67-year-old man with right diaphragm eventration. Superior bulging of the anterior right diaphragm is demonstrated on the coronal T2 TSE image (arrow). TSE: Turbo Spin Echo

Eleven of the cases included in the study were male (37.9%) and 18 (62.1%) were female. The mean age of the patients was 44.86 ± 17.57, ranging from 18 to 80 years. According to the evaluation of observer 1, 17 cases (58.6%) had normal findings, diaphragmatic weakness was detected in four cases (13.8%), diaphragmatic paralysis in four cases (13.8%), eventration of the diaphragm in two cases (6.9%), and diaphragmatic elevation due to pulmonary atelectasis in one case (3.4%). Observer 2 classified 16 cases (55.2%) as normal, six cases (20.7%) as diaphragmatic weakness, four cases (13.8%) as diaphragmatic paralysis, two cases (6.9%) as eventration of the diaphragm, and one case (3.4%) as diaphragm elevation due to pulmonary atelectasis. The static (Figure 2) and dynamic images (Video 1) of a case evaluated as diaphragmatic paralysis and the static (Figure 3) and dynamic (Video 2) images of another case classified as eventration of the diaphragm by both observers are presented as samples. The Kappa value was calculated as 0.889, indicating a strong agreement between the observers. Table 2 shows the classification of the cases according to the diagnosis of both observers.

**Video 1:** A 54-year-old woman with left diaphragm paralysis. The dynamic sBTFE-RLT sniffing video of the patient is shown in Figure 2 on the midline. Paradoxical upward movement of the left diaphragm dome during the inspiration is noted. sBTFE-RLT: Sense Balanced Turbo Field Echo Real-Time

**Video 2:** A 67-year-old man with diaphragm eventration. The dynamic sBTFE-RLT sniffing video of the patient in Figure 3 on the anterior line reveals normal diaphragmatic movements on both sides. sBTFE-RLT: Sense Balanced Turbo Field Echo Real-Time

**Table 2:** Diagnosis of the patients by two observers.

	Observer 1					
Observer 2		Normal	Weakness	Paralysis	Eventration	Lung atelectasis
	Normal	16	0	0	0	0
	Weakness	1	4	0	1	0
	Paralysis	0	0	4	0	0
	Eventration	0	0	0	2	0
	Lung atelectasis	0	0	0	0	1

## Discussion

Diaphragm dysfunctions, while often tolerated by patients, may require treatment due to respiratory distress and dyspnea [1]. Identifying underlying etiologies, including malignancies, is crucial, even if diaphragm dysfunction does not directly cause symptoms. The fluoroscopic sniff test has been the standard for demonstrating diaphragm dysfunction. Computed tomography is commonly used to detect underlying causes in cases of diaphragmatic paralysis or weakness.

This study evaluated the MRI protocol called bi-parametric diaphragm MR in which two sets of sequences were obtained that allow visualization of both the movement of the diaphragm and the etiology of dysfunction if any. The sBTFE-RLT sequence was used for dynamic evaluation, while the T2 TSE sequence was used for morphologic assessment. The high interobserver agreement in this study suggests the protocol's reliability in diagnosing diaphragmatic dysfunctions.

The observers were unable to reach a consensus on only two of the 29 cases. In one case, observer 1 classified it as normal diaphragmatic weakness, while observer 2 deemed it normal. In the second case, observer 1 evaluated it as diaphragmatic weakness, whereas observer 2 diagnosed it as eventration. These variations highlight the inherent subjectivity in diagnosing diaphragmatic conditions, which can be influenced by the observer's experience, interpretation of imaging, and subtle clinical presentations. Diaphragmatic weakness, in particular, is a condition that can present with varying degrees of severity and can be difficult to differentiate from other diaphragmatic abnormalities without standardized criteria. Therefore, incorporating quantitative assessment methods in future studies could enhance diagnostic accuracy and reduce observer variability, providing more robust and reliable diagnoses in such cases.

Previous studies have explored MRI of the diaphragm. Shanmuganathan et al. used MRI in the evaluation of diaphragmatic injury in 16 acute blunt trauma cases. However, the authors did not assess diaphragmatic dysfunctions and movements; they only obtained static sequences to monitor the diaphragm integrity [8]. Taylor et al. utilized real-time MRI navigator echoes to monitor diaphragm movement. Initially designed to reduce breathing-related artifacts in upper abdominal MRI exams, it was found to provide significant results in showing diaphragm movements, as well [4]. Apart from these two studies from the literature, another research was undertaken between 1999 and 2002 to perform MRI to examine the diaphragm and chest wall movements in emphysema cases and found significant changes [3,9]. In 2000, Cluzel et al. undertook a quantitative assessment of the movement of the diaphragm and rib cage during breathing based on 3D reconstructed images [10]. In the following years, researchers compared quantitative values obtained​​ from MRI examinations with those of the spirometric respiratory function tests [11] and utilized MRI in the evaluation of the diaphragm in Pompe disease [12]. In fact, the role of MRI in assessing diaphragm movement is known and occasionally used in the clinic; e.g., for the demonstration of pleural invasion [13].

In subsequent years, studies on diaphragm imaging with MRI have increasingly focused on the relationship between COPD and diaphragm function. These studies have found that diaphragm movements in COPD patients, as observed through MRI, are significantly reduced compared to healthy control groups [14-16].

In addition to fluoroscopy, ultrasonography can also be radiologically used for the evaluation of diaphragm movement. Ultrasonography does not contain ionizing radiation and is practical in terms of ease of access [1,17-19]; however, it has certain disadvantages, such as the inability to visualize the lungs, the instantaneous evaluation of the image depending on the person, and the difficulty of assessing paradoxical motion due to the challenges in displaying the two parts of the diaphragms at the same time in paralysis cases. Nevertheless, the wide availability and low cost of ultrasonography may bring this method to the forefront as the initial examination technique.

The evaluation of diaphragmatic dysfunctions with the combined application of both static and dynamic sequences named bi-parametric MRI has supplied high interobserver agreement. The imaging protocol used in this study gave satisfactory results and can be improved with further studies. MRI also has certain disadvantages, including its limited availability, being difficult to use, and its high cost. However, considering that MRI does not require ionizing radiation and has a role in resolving diagnostic problems only in one step, it may be predicted that MRI has the potential to be used as an advanced examination technique and even to replace fluoroscopy in the future.

The limitations of our study include the focus solely on interobserver variability, the small number of observers (only two), the lack of comparison with a gold standard diagnostic test, and the absence of sensitivity and specificity calculations. Additionally, our sample size is relatively small. This study serves as an initial experience in this area, highlighting the need for further advanced studies with larger sample sizes and comparisons using gold-standard tests.

## Conclusions

Our initial experience with bi-parametric MRI demonstrates that this technique is a promising tool for evaluating diaphragmatic dysfunctions. Bi-parametric MRI of the diaphragm might represent a significant advancement in the imaging of the diaphragm, providing a reliable and comprehensive method for diagnosing diaphragmatic pathologies such as diaphragmatic paralysis, eventration, and weakness with high interobserver agreement. Further studies are needed with a larger number of patients, comparing bi-parametric MRI with imaging methods used in routine practice.
